# Effects of different diets used in diet-induced obesity models on insulin resistance and vascular dysfunction in C57BL/6 mice

**DOI:** 10.1038/s41598-019-55987-x

**Published:** 2019-12-20

**Authors:** Philipp Lang, Solveig Hasselwander, Huige Li, Ning Xia

**Affiliations:** Department of Pharmacology, Johannes Gutenberg University Medical Center, Mainz, Germany

**Keywords:** Cardiovascular biology, Cardiovascular diseases

## Abstract

The aim of the present study was to compare different diets used to induce obesity in a head-to-head manner with a focus on insulin resistance and vascular dysfunction. Male C57BL/6J mice were put on standard chow diet (SCD), normal-fat diet (NFD), cafeteria diet (CAF) or high-fat diet (HFD) for 12 weeks starting at the age of 6 weeks. Both CAF and HFD led to obesity (weight gain of 179% and 194%, respectively), glucose intolerance and insulin resistance to a comparable extent. In aortas containing perivascular adipose tissue (PVAT), acetylcholine-induced vasodilation was best in the NFD group and worst in the CAF group. Reduced phosphorylation of endothelial nitric oxide synthase at serine 1177 was observed in both CAF and HFD groups. Plasma coagulation activity was highest in the HFD group and lowest in the SCD group. Even the NFD group had significantly higher coagulation activity than the SCD group. In conclusions, CAF and HFD are both reliable mouse diets in inducing visceral obesity, glucose intolerance and insulin resistance. CAF is more effective than HFD in causing PVAT dysfunction and vascular dysfunction, whereas hypercoagulability was mostly evident in the HFD group. Coagulation activity was higher in NFD than NCD group.

## Introduction

According to the World Health Organization, 39% of adults aged 18 years and older were overweight and 13% were obese in 2016^1^. This means that obesity has nearly tripled worldwide since 1975 and therefore can be termed a growing global epidemic^1^.

Obesity is associated with a general dysregulation of metabolic homeostasis, resulting in insulin resistance, dyslipidemia, altered regulation of blood pressure, and increased risk for diabetes, cardiovascular disease, chronic kidney disease, and cancer^2^. Therefore, obesity and its comorbidities represent a major field of interest for basic science and clinical research.

The obesity epidemic results from an imbalance of food intake, basal metabolism and energy expenditure, with increased energy intake or decreased physical activity being the most important factors^3^. Especially the alterations in Western-style diets due to changes in availability, quality, quantity and source of consumed food are leading causes for growing obesity^4^.

Since complications from obesity such as diabetes and cardiovascular disease usually require decades to develop, surrogate animal models are indispensable for studying the molecular aspects of obesity and its pathophysiological effects^5^. Counted among those are rodent models such as genetic loss-of-function mutations, transgenic gain-of-function mutations, polygenic models and different environmental exposure models^5–7^. Here diet-induced obesity (DIO) models represent the best fit for comparison to human obesity related pathologies^5–7^.

The C57BL/6J mouse model has been shown to be a particularly good model mimicking human metabolic disorders that are observed in obesity^5,7^. When fed *ad libitum* with a high-fat diet (HFD), obesity, hyperinsulinemia, hyperglycemia and hypertension establish in these mice. Control groups fed *ad libitum* with normal chow however, do not develop any metabolic abnormalities^5,8^.

Diverse high energy diets have been utilized to induce obesity and related metabolic disorders in rodent models, though the dietary mediation has not been absolutely standardized^9^. Traditionally, these diets consist of a simple exchange of carbohydrate-derived calories with fat-derived calories and are compared to a standard chow diet (SCD) as control. Though, this poses the problem that HFD compared to the control exhibits marked differences in micro- and macronutrient content^10^. Therefore, obtained results cannot be accurately attributed to HFD only. A study from 2008 showed that of the 35 papers examined only five papers compared diets using identical nutrients differing only in relative amounts of fat and carbohydrate^10,11^. Furthermore SCDs show different nutrient contents from batch to batch^12^. Commonly chow diets are grain-based and supplemented with fats, vitamins and minerals^13^. Every ingredient is rich on nutrients but also biologically active non-nutrients like heavy metals and phytoestrogens. The nutrient levels are naturally subject of change and therefore their exact content is hard to define^14^. So, reproducibility as well as publication external comparability is limited^13^. Nevertheless, so far only few researchers switched from SCD to a purified ingredient, HFD-matching control diet like normal-fat diet (NFD)^12^. Purified ingredient diets consist of highly refined ingredients with almost no non-nutrient supplements. The content therefore is consistent and highly controllable^14^. However, it is not definitely established yet whether purified control diets are really superior to SCDs. While some publications state no difference between NFD and SCD^15^, others prefer NFD over SCD^12^ and *vice versa*^16^.

Another complication posed by HFD in mouse models is that HFD does not accurately reflect an obese human’s diet^11,17^. Therefore, the cafeteria diet (CAF) model was introduced. In addition to normal chow, mice are fed *ad libitum* with a variety of highly palatable, high-salt, high-fat and low-fiber, energy dense foods accessible in Western societies, which are associated with snacking and weight gain^4,6^. It was shown that this hedonic feeding promotes voluntary hyperphagia, rapid weight gain, increased fat pad mass and glucose and insulin intolerance^4,6,18,19^. In comparison to HFD, CAF was even considered a more robust model for metabolic syndrome^4,6,18^. To date, however, the differences between the CAF model and other DIO animal models have not been sufficiently examined to allow an explicit and clear decision making for which to choose for certain experiment settings. Although a few previous studies have addressed the difference of the diets on adiposity and inflammation^4,18^, no study has directly compared the effects of the diets on vascular function or coagulation. The aim of the present study was to compare the different diets in a head-to-head manner as well as to identify the ideal control diet for studies regarding vascular function as well as insulin resistance.

## Material and Methods

### Animals and diets

Male C57BL/6J mice were obtained from Harlan Laboratories, Roßdorf, Germany. SCD (V1126), NFD (E15748-04) and HFD (E15744-34, corresponding to Research Diets D12451) were from ssniff Spezialdiäten GmbH, Soest, Germany. The HFD was a defined, lard-based diet with 45 kJ% energy from fat, 20 kJ% from protein and 35 kJ% from carbohydrates. The NFD was a defined control diet with identical nutrients as HFD differing only in relative amounts of fat and carbohydrate (13 kJ% from fat, 20 kJ% from protein and 67 kJ% from carbohydrates). The mice were put on SCD, NFD, CAF or HFD for 12 weeks starting at the age of six weeks. Food was exchanged and weighed twice a week, except for the CAF cages, which had their food changed and weighed daily. Each cage housed five animals. All the animals were weighed once a week. For calculation of the caloric uptake, the consumed food in gram was multiplied with the calories per gram of the respective type of food. In case of the CAF-fed mice, every type of food was separately weighed and multiplied with the respective amount of calories per gram. Mice in the SCD-, NFD- and HFD-groups had their respective diet offered *ad libitum* (Table 1). CAF-mice were provided with standard chow and a selection of daily changing two snacks with different taste (usually salty or sweet) offered *ad libitum*. The used snacks and their nutrition facts are listed in Table 2.

**Table 1:** Nutrition facts of the SCD, NFD and HFD.

Diet		SCD		NFD		HFD	
		%	cal/g	%	cal/g	%	cal/g
Fat	kJ%	11	36.52	13	46.93	45	207.45
	Crude fat	4.5	1.64	5.1	2.39	23.60	48.96
	SFA	0.73	0.27	0.79	0.37	8.66	17.97
	MUFA	1	0.37	1.29	0.61	10.04	20.83
	PUFA	2.73	1	2.94	1.38	3.83	7.95
Protein	kJ%	36	119.52	20	72.2	20	92.2
	Crude protein	22.1	26.41	18.2	13.14	22.0	20.28
Carbohydrate	kJ%	53	175.96	67	241.87	35	161.35
	Crude fiber	3.9	6.86	8.0	28.8	5.7	9.20
	Starch	35.8	62.99	37.3	90.21	6.8	10.97
	Sugar	5.2	9.15	11	26.61	21.1	34.04
Sodium (%)		0.25		0.20		0.20	
Energy (kcal/g)		3.32		3.61		4.61	
Consumed (g/day)		3.26		2.76		2.66	

SFA, saturated fatty acids; MFA, monounsaturated fatty acids; PUFA, polyunsaturated fatty acids.

**Table 2:** Nutrition facts of the Cafeteria snacks.

	Fat		Protein		TotalCarb.		Fiber		Sugar		Sodium		Energy (kcal/g)	Consume (g)
	%	cal/g	%	cal/g	%	cal/g	%	cal/g	%	cal/g	%	cal/g		
Kinder chocolate	34.4	194.02	8.8	49.63	54.7	308.51	1.1	6.20	52.5	296.1	0.12	0.68	5.64	1.73
Nougat waffles	33.4	182.36	9.8	53.51	53.7	293.20	3.3	18.02	34.4	187.82	0.2	1.09	5.46	1.42
Chocolate rice waffles	29.1	155.10	6.4	34.11	62.3	332.06	1.8	9.59	48.0	255.84	0.06	0.32	5.33	1.16
Mini Cake	28.0	142.24	4.7	23.88	58.0	294.64	4.0	20.32	29.0	147.32	0.5	2.54	5.08	1.43
Lion bar	22.9	112.9	5.5	27.12	66.8	329.32	1.3	6.41	53.1	261.78	0.13	0.64	4.93	0.87
Cocos chocolate bar	24.6	120.05	4.1	20.01	59.4	289.87	1.6	7.81	47.3	230.82	0.11	0.54	4.88	0.98
Double cheese sticks	27.0	135.27	16.0	80.16	52.8	264.51	3.8	19.03	1.4	7.01	1.8	9.018	5.01	0.24
Mini Salami	45.0	229.5	25.0	127.5	1.0	5.1	0.1	0.51	0.9	4.6	1.58	8.058	5.10	0.98
Pretzel Pieces	27.3	141.41	8.5	44.03	58.5	303.03	0.25	1.30	3	15.54	2.2	11.396	5.18	0.56
Cheese Cubes	29.0	103.53	24.0	85.68	0.2	0.71	0.1	0.36	0.1	0.36	0.33	1.18	3.57	1.69
Cocos flocks	62.0	385.64	7.8	48.52	21.9	136.22	13.7	85.21	4.8	29.86	0	0	6.22	0.78
Cheese Sticks	24.0	117.84	12	58.92	55.0	270.05	0	0	2	9.82	0.92	4.52	4.91	1.04
Chocolate	31.2	173.47	6.3	35.03	58.2	323.59	0	0	56.5	314.14	0.3	1.67	5.56	0.95
Crackers	23.0	111.78	8.0	38.88	62.1	301.81	2.1	10.21	10.0	48.6	0.95	4.62	4.86	0.60
Cheese	34.0	142.12	28.0	117.04	<0.1	<0.41	<0.1	<0.41	<0.1	<0.41	1.6	6.69	4.18	1.87

Nougat waffles: Storck Knoppers; Chocolate rice waffles: Rübezahl Sun Rice; Mini cake: Nestlé Yes!; Cocos chocolate bar: Mars Inc. Bounty; Double cheese sticks: DeBeukelaer CheeOps; Mini salami: Jack Link’s BiFi; Pretzel Pieces: Snyder’s Pretzel Pieces cheddar cheese flavour; Cheese Cubes: Grünwalder Käsewürfel mild & nussig; Cocos flocks: Alnatura Cocos Chips; Cheese Sticks: Ja! Käsegebäckstangen; Chocolate: Ja! Alpensahneschokolade; Crackers: Lorenz Clubs Cracker; Cheese: Rewe Bio Bergkäse. The shown values in the last column are average amounts of snacks consumed per mouse per day.

The animal experiment was approved by the responsible regulatory authority (Landesuntersuchungsamt Rheinland-Pfalz; 23 177-07/G 12-1-021 & G 17-1-020) and was conducted in accordance with the German animal protection law, the EU Directive 2010/63/EU for animal experiments and the National Institutes of Health (NIH) Guide for the Care and Use of Laboratory Animals.

### GTT and ITT

Glucose tolerance tests (GTT) and insulin-tolerance tests (ITT) were performed in the 12^th^ week of experimental feeding. Mice were fasted overnight and weighed before measurement. Blood was obtained from the tail vein by cutting off 2 mm of the tip with a sterile single use Feather scalpel. For GTT, 1 g of glucose per kilogram bodyweight was injected intraperitoneally in form of a 10% glucose solution. The blood glucose level was measured before and 15, 30, 60, 90 and 120 minutes after the administration of the glucose solution. For ITT, 1 IU per kg bodyweight of human insulin (Humulin, Lilly Deutschland GmbH in 10 ml ampoule with 100 IU/ml) was injected intraperitoneally. The insulin stock solution was diluted 1:1000 with Dulbecco’s phosphate buffered saline (Sigma Life Science) resulting in a 0.1 IU/ml solution. The blood glucose level was measured before and 15, 30, 60, 90 and 120 minutes after the insulin injection. Measurements were performed with the Bayer Contour glucose meter.

### Tissue collection

Tissue collection was performed after the 12^th^ week of experimental feeding. Mice were fasted overnight and fasting glucose was measured. Fasting glucose was measured by cutting the tail tip. Additional 50 µl blood was collected from the cut tail tip to generate serum for insulin measurement. Anesthesia was performed with Forene 100% isoflurane (Abbot GmbH & Co. KG) and the thorax was opened under isoflurane anesthesia. Blood was collected by cardiac puncture. Approximately 500 µl blood was collected from each mouse and anticoagulated with 3.8% sodium citrate (blood to sodium citrate is 9:1 in volume). The anticoagulated blood was centrifuged (2000 g, 20 min) at room temperature to generate plasma. Liver, thoracic aorta including the perivascular adipose tissue (PVAT), epidydimal fat pads, retroperitoneal fat pads, intestine with mesenteric artery and PVAT were isolated. Fat pads were weight and instiantly frozen in liquid nitrogen. Aorta and intestine were put into ice-cold Krebs buffer solution for further preparation. Thoracic aorta was used for used for myograph experiments and the rest was separated from the PVAT and frozen in liquid nitrogen. PVAT was frozen separately.

### Myograph experiments

For vascular function studies, thoracic aorta was dissected into rings of 2–3 mm in length. Aortic PVAT and connective tissues were either removed or left intact, respectively. Isometric tension was recorded using a wire myograph system (Danish Myo Technology, Aarhus, Denmark). The rings were equilibrated for 60 min and contracted two times with 120 mmol/L KCl. For assessment of vascular function, the rings were pre-contracted with noradrenaline to reach the submaximal tension (80% of that obtained with 120 mmol/L KCl), before vasodilatation was induced by acetylcholine^20,21^.

### ***Ex vivo*** coagulation assays

The activated partial thromboplastin time (aPTT), prothrombin time (PT) and the funcational activity of the coagulation factors were tested on the semi‐automated Amelung KC4 Delta micro-coagulation analyzer (Trinity Biotech GmbH, Lemgo, Germany). All reagents for clotting assays were from Siemens Healthcare Diagnostic Products GmbH (Marburg, Germany) and were prepared according the manufacturer’s instructions.

For PT measurment, 100 µl citrate plasma was added to the coagulation cuvette containing a metal bead and incubated for 60 seconds at 37 °C in the coagulometer while the metal bead was stirring. Then, 100 µl prewarmed PT reagent Thromborel S (containing thromboplastin and CaCl_2_) was added to start clotting. The time to the formation of a clot was recorded^22–24^.

For APTT measurement, 100 µl citrate plasma was added to the coagulation cuvette containing a metal bead and the sample was warmed to 37 °C. Then, 100 µl pre-warmed APTT reagent Pathromtin SL (containing silicon dioxide particles and plant phospholipids) was added and incubated for 5 min at 37 °C while metal bead was stirring. The reaction was then triggered by the addition of 100 µl of warmed 25 mmol/L CaCl_2_ and the clot time was recorded^22–24^.

The functional activity of coagulation factors was studied with assays modified from human clinical assays by mixing mouse plasma with factor-deficient human plasma^23^. For factors FII, FV, FVII and FX, 100 µl of diluted plasma samples (1:20 diluted in imidazole buffer) were added to 100 µl pre-warmed (37 °C) factor-deficient plasma followed by an incubation for 60 seconds. Then, 200 µl of pre-warmed PT reagent was added and the clotting time recorded. For factors FVIII, FIX, FXI, and FXII, 100 µl of the diluted plasma samples (1:5 diluted in imidazole buffer) and 100 µl of the APTT reagent were added to 100 µl pre-warmed deficient plasma. The clot time was recorded after adding 100 µl of warmed 25 mmol/L CaCl_2_^22–24^.

### Western blot

Protein samples (30 µg each) were separated on a Bis-Tris gel and transferred to a nitrocellulose membrane. After the transfer to a nitrocellulose membrane, the blots were blocked in 5% milk powder in TBST (10 mmol/L Tris-HCl, pH 7.4, 150 mmol/L NaCl with 0.1% Tween 20) for one hour at room temperature. The primary antibodies were diluted in the same solution at 4 °C over night. The following primary antibodies were used: rabbit monoclonal antibody against phospho-eNOS at Ser1177 (catalog number 9571, Cell Signaling; 1:1000), mouse monoclonal antibody against eNOS (catalog number 610297, BD Bioscience; 1:2000), rabbit monoclonal antibody against phospho-Akt Ser473 (catalog number 4060, Cell Signaling; 1:2000), rabbit monoclonal antibody against Akt (catalog number 4691, Cell Signaling; 1:1000), mouse monoclonal antibody against β tubulin I (catalog number T8328, Sigma Aldrich; 1:5000). Subsequently, blots were washed with TBST and incubated for one hour with a horseradish peroxidase-conjugated secondary antibody diluted in 5% milk in TBST. After washing in TBST and TBS, the immunocomplexes were visualized using an enhanced horseradish peroxidase/luminol chemiluminescence reagent (PerkinElmer Life and Analytical Sciences, Boston, MA) according to the manufacturer’s instructions. Densitometric analysis of scanned blots was performed using the Quantity One software (Bio-Rad).

### Measurement of fasting insulin

Insulin was measured with the enzyme-linked immunosorbent assay (ELISA) using the Mercodia Ultrasensitive Mouse Insulin ELISA kit (Mercodia AB, Uppsala, Sweden) and 10 µl serum samples according to the manufacturer’s instructions.

### Statistics

Results are expressed as mean ± SEM (standard error of the mean). Two-tailed, unpaired Student’s t test was used for comparison of two groups. One-way analysis of variance (ANOVA) followed by Fisher’s protected least significant difference test was performed to compare more than two groups. Curves were analyzed by either ANOVA or by calculation of the area under the curve (AUC) with the assumption that all events are above zero. P values <0.05 were considered significantly different. Statistical analysis was performed with GraphPad Prism 7.02 (GraphPad Software, La Jolla, CA, USA).

### Ethics approval and consent to participate

The animal experiment was approved by the responsible regulatory authority (Landesuntersuchungsamt Rheinland-Pfalz; 23 177-07/G 12-1-021). Animal care and experiments conform to the German animal protection law and the Directive 2010/63/EU of the European Parliament on the guidelines for the protection of animals used for scientific purposes.

## Results

### Food consumption, energy intake and weight gain

The energy and nutrition intake of the CAF group were calculated by analyzing the daily snack and chow consumption. The CAF mice took approximately 84.8% of their energy from snacks and 15.2% from chow. As a result, 52.6% of their energy was from fat, compared to 45% fat-derived calories in the HFD group (Table 3).

**Table 3:** Nutrition Facts and consumption of the diets.

	SCD		CAF		NFD		HFD	
	kcal%	kcal/g	kcal%	kcal/g	kcal%	kcal/g	kcal%	kcal/g
Fat	11	0.37	52.6	2.33	13	0.47	45	2.07
Carbohydrate	53	1.76	31.2	1.38	67	2.14	35	1.61
Protein	36	1.20	16.2	0.72	20	0.72	20	0.92
Water intake (ml/day)	3.41		2.53		3.19		2.52	
Food intake (g/day)	3.26		2.84		2.76		2.66	
Energy intake (kcal/day)	10.82		12.56		9.96		12.26	

The nutrition facts of CAF are calculated based on the amount of consumed chow and snacks. Water, food and energy intakes shown are average values per mouse per day.

**Figure 1:**
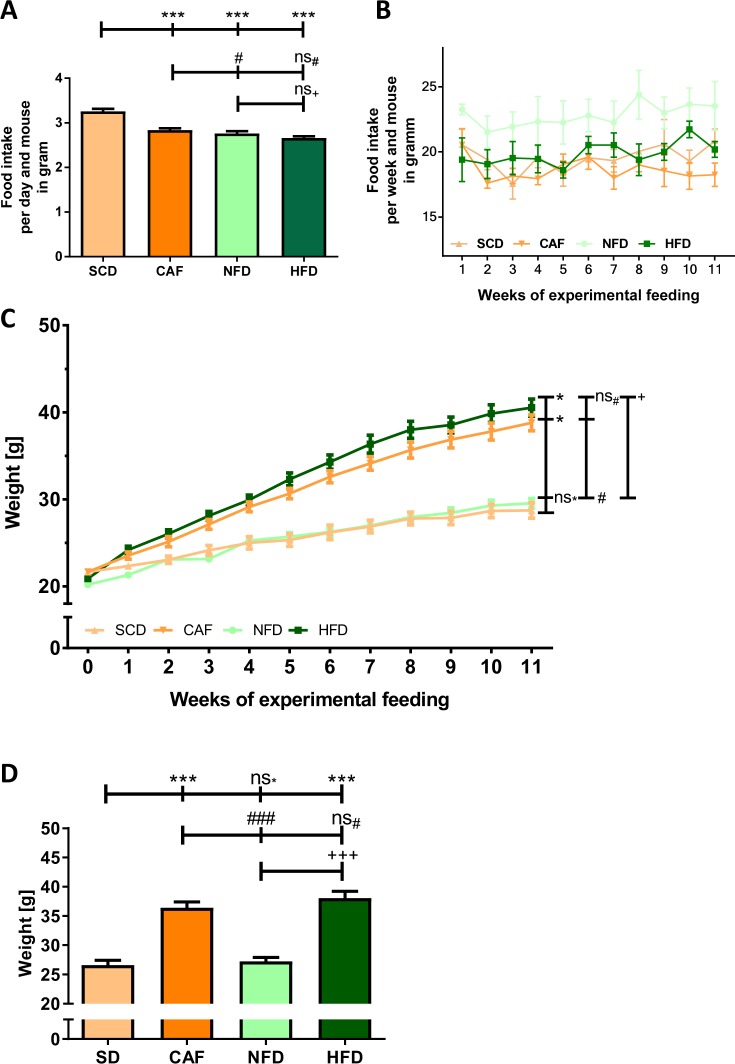
Development of the bodyweight. Male C57BL/6J mice were put on standard chow diet (SCD), cafeteria diet (CAF), normal-fat diet (NFD) or high-fat diet (HFD) at the age of 6 weeks for another 12 weeks. Food intake per day per mouse in gram (**A**) and changes in food consumption per week per mouse in gram over the feeding period (**B**) are pictured. ANOVA followed by Fisher’s protected least significant difference test as *post hoc* method was performed to compare all four groups. Panel C shows the development of the bodyweight over time. Panel D shows the bodyweight after 12 weeks of experimental feeding. Significances marked with ^*,#^ and ^+^refer to comparisons to the SCD, CAF and NFD group, respectively. ^*,#^P < 0.05; ^***,###,+++^P < 0.001; ns, not significant. n = 15 (SCD and CAF) or 20 (NFD and HFD), respectively.

The food consumption in gram was lower in NFD (3.26 g per day per mouse), HFD (2.84 g per day per mouse) and CAF (2.76 g per day per mouse) groups compared to SCD (2.66 g per day per mouse) (Table 3, Fig. 1A). Transferring food consumption to energy intake showed that both DIO feeding groups had a significantly higher energy intake compared to both control groups (Table 3). There were no significant differences between the caloric intake of HFD and CAF or between SCD and NFD (Table 3). The amount of food intake did not change in any group over the time of experimental feeding (Fig. 1B).

After twelve weeks of experimental feeding, animals in both DIO groups, HFD and CAF, displayed a significantly increased body weight compared to the control groups, SCD and NFD (Fig. 1C,D). There was no significant difference of weight gain between HFD and CAF, nor between SCD and NFD. The body mass in the HFD and CAF groups became significantly higher than the controls after three and four weeks of feeding, respectively.

**Figure 2:**
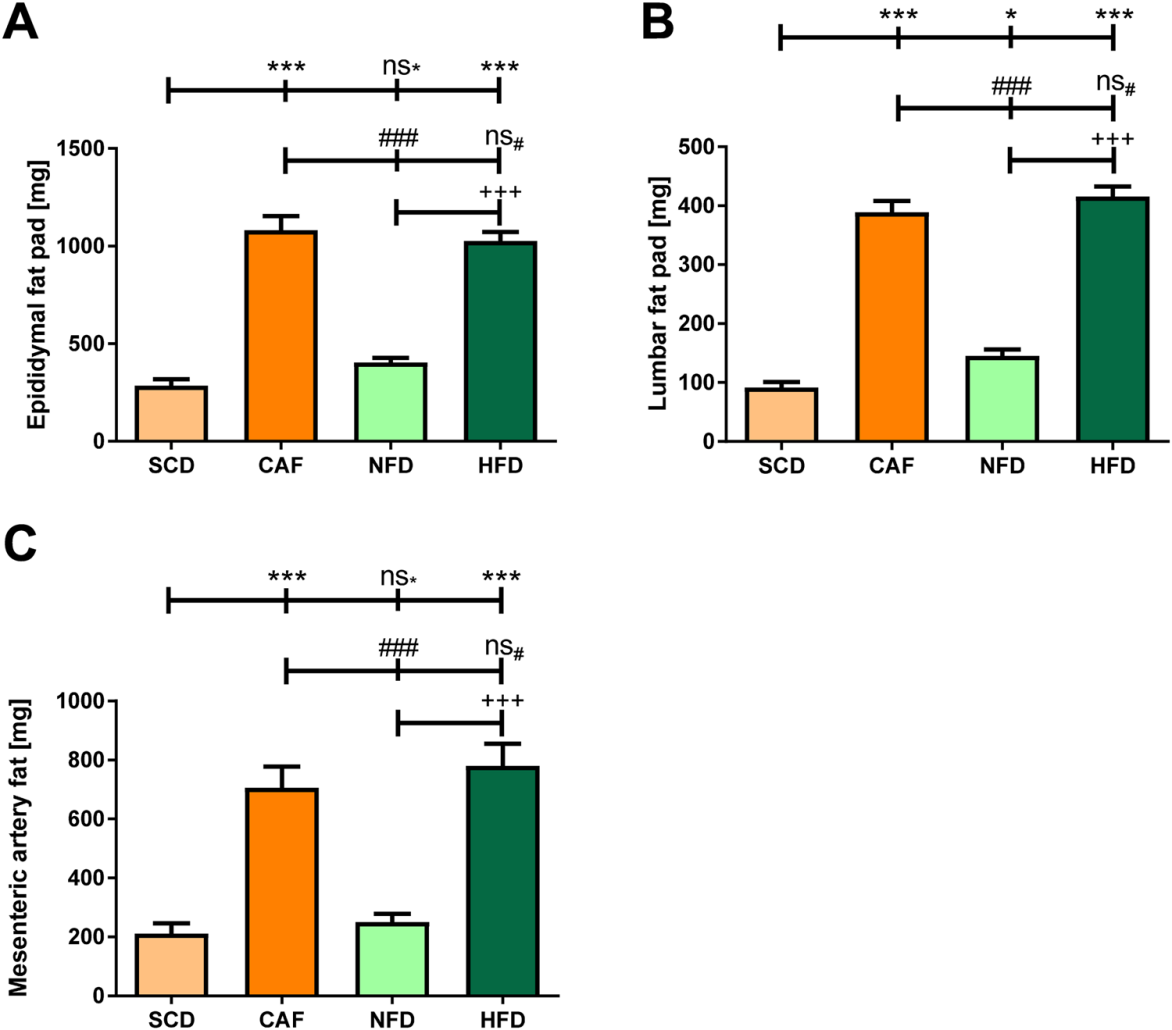
Weight of fat mass. Male C57BL/6J mice were put on experimental diets at the age of 6 weeks for another 12 weeks. The weights of epididymal (**A**), lumbar (**B**) and mesenteric fat pads (**C**) at the end of the experiment are shown. Significances marked with ^*, #^ and ^+^refer to comparisons to the SCD, CAF and NFD group, respectively *P < 0.05; **P < 0.01; ^***###,+++^P < 0.001; ns, not significant. n = 15 (SCD and CAF) or 20 (NFD and HFD), respectively.

Regarding adiposity, DIO groups (HFD and CAF) had significantly higher epididymal fat (Fig. 2A), lumbar fat (Fig. 2B) and mesenteric fat (Fig. 2C) masses than the controls (SCD and NFD). No difference between HFD and CAF was found. SCD and LFD groups were comparable in epididymal, lumbar and mesenteric fat mass. The lumbar fat mass of NFD-fed mice was slightly higher compared to SCD-fed mice.

### Fasting glucose, insulin, GTT and ITT

**Figure 3:**
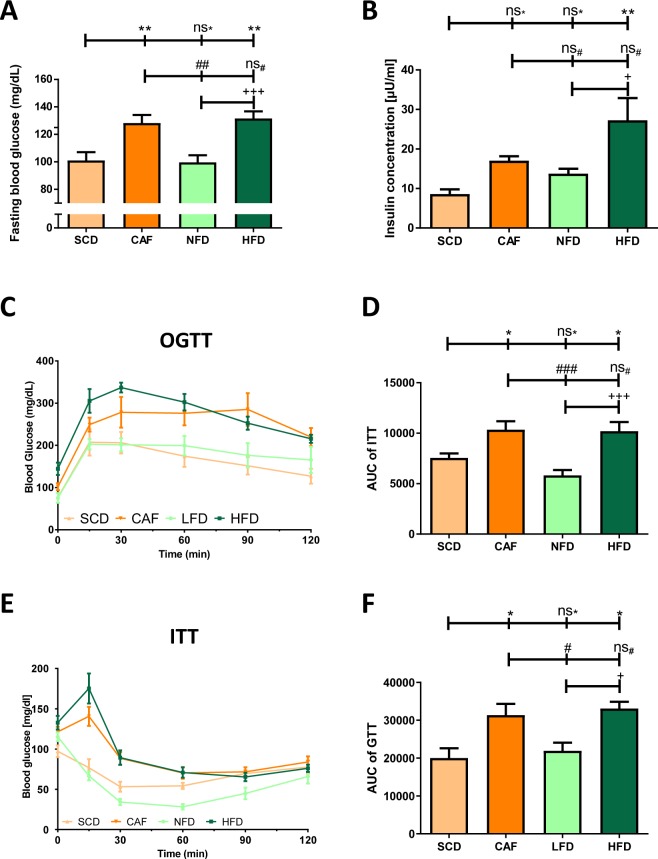
Glucose intolerance. Male C57BL/6J mice were put on experimental diets at the age of 6 weeks for another 12 weeks. Panel A shows the fasting glucose levels in the 12th week of feeding. n = 15 (SCD and CAF) or 20 (NFD and HFD), respectively. Panel B shows the plasma insulin concentration in the 12th week of feeding. n = 6 (SCD, NFD and HFD) or 5 (CAF), respectively. Panel C shows the results of GTT at the end of the experiment after overnight fasting. n = 5 (SCD, CAF and HFD) and 9 (NFD), respectively. Panels E shows the results of ITT (1 IU/kg body weight insulin was injected intraperitoneally) in the 12^th^ week of experimental feeding after overnight fasting. n = 10 (SCD and NFD) and 9 (CAF and HFD), respectively. The area under the curve (AUC) of GTT and ITT are shown in panels D and F, respectively. Significances marked with ^*,#^ and ^+^refer to comparisons to the SCD, CAF and NFD group, respectively. ^*,#,+^P < 0.05, ^**,##^P < 0.01, ^***,###,+++^P < 0.001, ns, not significant.

Fasting glucose levels were significantly higher in the DIO groups (HFD and CAF) than the controls (Fig. 3A). HFD mice had also significantly higher blood levels of insulin (Fig. 3B). GTT analysis showed significantly increased blood glucose values two hours after glucose injection in both DIO groups compared to both control groups (Fig. 3C). The area under the curve was greater in both DIO groups than in both control groups, indicating an impaired glucose tolerance in HFD- and CAF-fed mice (Fig. 3D).

Both DIO groups also displayed higher blood glucose levels in ITT. The 15- and 30-minutes blood glucose levels were significantly higher in HFD- and CAF-fed mice than in both of the control groups (Fig. 3E). The area under the curve was significantly greater in both experimental groups compared to SCD- and NFD-fed mice, indicating insulin resistance in these groups (Fig. 3F).

### Vascular function

**Figure 4:**
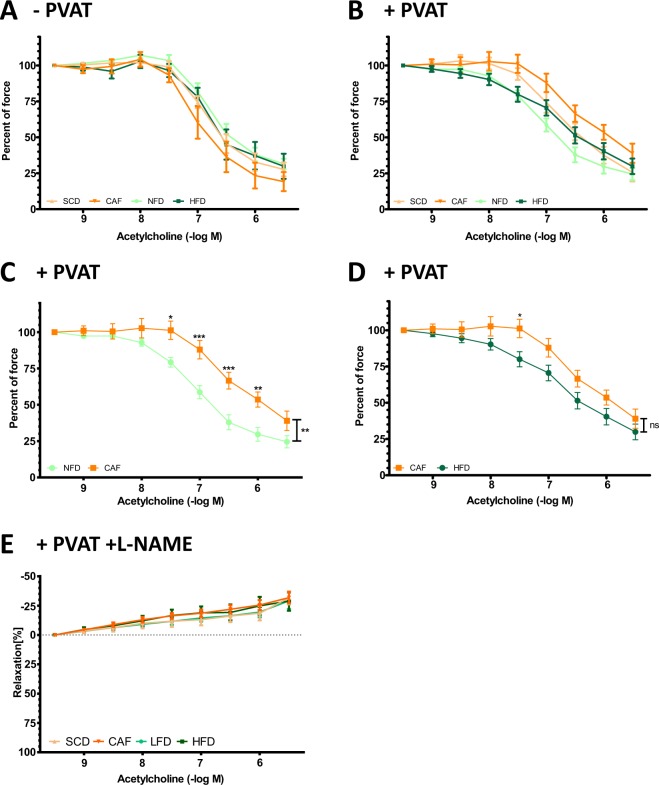
Vascular function. Male C57BL/6J mice were put on experimental diets at the age of 6 weeks for another 12 weeks. Vasodilation was induced by acetylcholine in norepinephrine-pre-contracted aorta in myograph experiments, without PVAT (**A**), with PVAT (**B**–**E**), or in the presence of NO synthase inhibitor L-NAME (**E**). *P < 0.05, **P < 0.01, ***P < 0.001. n = 9 (SCD and CAF) and 15 (NFD and HFD), respectively.

In myograph experiments with PVAT-free aorta, no difference was observed in acetylcholine-induced vasodilation (Fig. 4A). This is consistent with our previous studies^20,21^. In contrast, significant difference in vascular function was evident, if the PVAT is left intact. The best vasorelaxation was seen in NFD and the worst in CAF with highly significant difference between the two groups (Fig. 4B,C). The vasodilation in CAF aorta was weaker than that of HFD at the acetylcholine concentration of 30 nM (Fig. 4D). The difference between SCD and CAF was not significant, nor that between NFD and HFD. The relaxation induced by acetylcholine was completely blocked by the eNOS inhibitor L-NAME (Fig. 4E), consistent with the concept that acetylcholine-induced vasodilation of the mouse aorta (irrespective of the presence of PVAT) is completely NO-dependent^20,21^.

### Phosphorylation of eNOS

**Figure 5:**
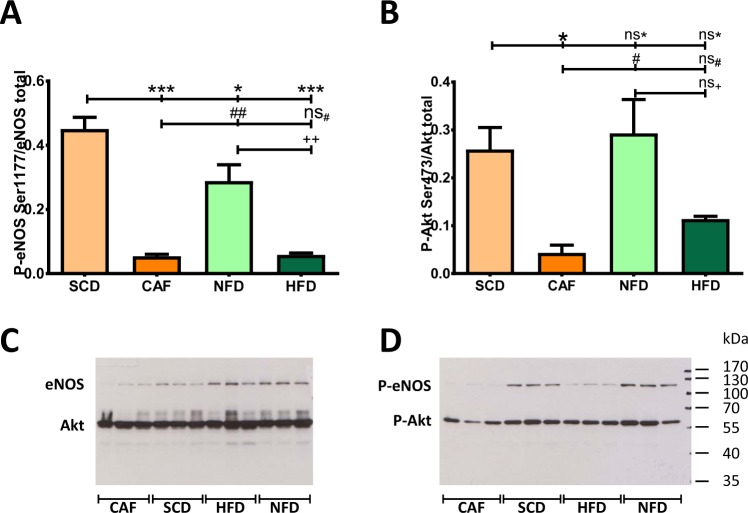
Phosphorylation of eNOS. Male C57BL/6J mice were put on experimental diets at the age of 6 weeks for another 12 weeks. Phosphorylation of eNOS at Ser1177 (**A**) and of Akt at Ser473 (**B**) in aortic PVAT was studied by Western blot analyses. The blots shown are representative of four independent experiments with similar results. Significances marked with ^*,#^ and ^+^refer to comparisons to the SCD, CAF and NFD group, respectively. ^*,#^P < 0.05, ^**,##,++^P < 0.01, ***P < 0.001, ns, not significant. n = 12.

Reduced eNOS serine 1177 phosphorylation in PVAT was observed in both HFD and CAF groups (Fig. 5A), which was associated with a reduced Ser-473 phosphorylation of the upstream kinase Akt (Fig. 5B).

### Coagulation

**Figure 6:**
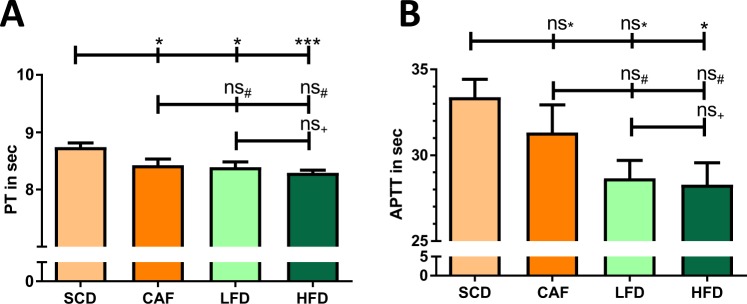
Coagulation. Male C57BL/6J mice were put on experimental diets at the age of 6 weeks for another 12 weeks. Prothrombin time (PT) (**A**) and activated partial thromboplastin time (APTT) (**B**) were measured with citrate plasma on a KC4 Delta Amelung Coagulometer. Significances marked with ^*,#^ and ^+^refer to comparisons to the SCD, CAF and NFD group, respectively. *P < 0.05, ***P < 0.001, ns, not significant. n = 10 (SCD and CAF), 9 (NFD) and 11 (HFD), respectively.

**Figure 7:**
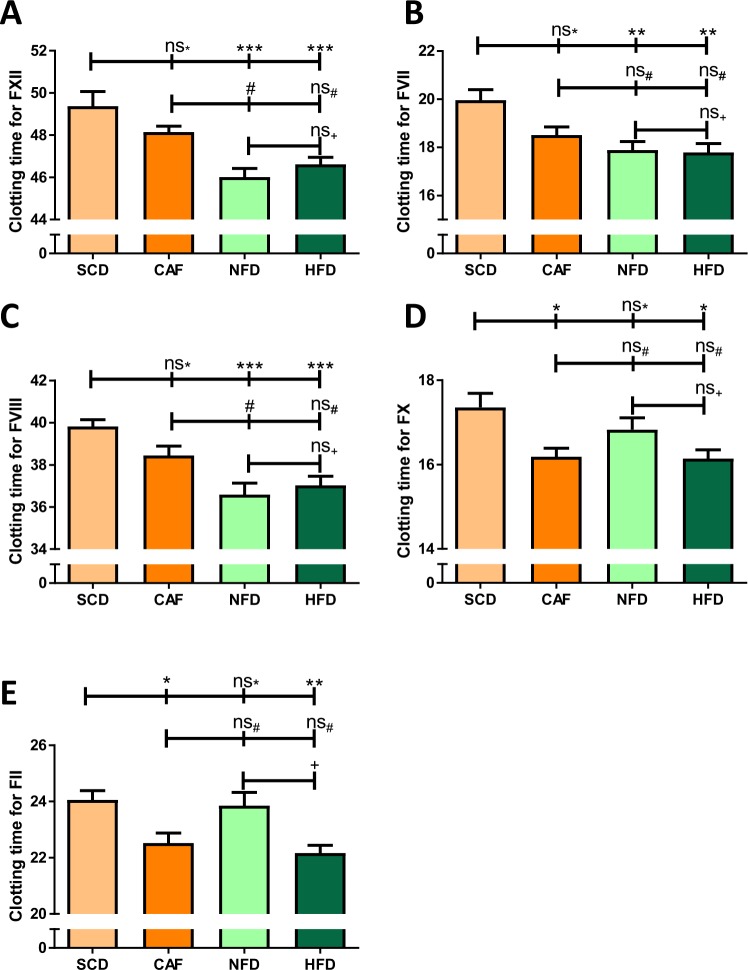
Activity of coagulation factors. Male C57BL/6J mice were put on experimental diets at the age of 6 weeks for another 12 weeks. The activity of coagulation factors was determined using deficient plasma (shorter coagulation time means higher activity) on a KC4 Delta Amelung Coagulometer. Shorter coagulation time indicates higher activity. Significances marked with ^*,#^ and ^+^refer to comparisons to the SCD, CAF and NFD group, respectively. ^*,#,+^P < 0.05, **P < 0.01, ***P < 0.001, ns, not significant. n = 9 (SCD, NFD and CAF) and 11 (HFD), respectively.

Compared to SCD, all other groups, even NFD, showed shortened PT (Fig. 6A). A similar trend was observed in APTT, although only the difference between HFD and SCD was statistically significant (Fig. 6B). Regarding the individual coagulation factors, no differences in the functional activity of the FV, FIX and FXI were found between the groups (data not shown). The activities of FXII, FVIII, FVII, FX and FII were lowest in the SCD group, and were significantly enhanced in the HFD group (Fig. 7). Compared to the SCD group, CAF mice had enhanced activity of FX and FII, whereas the NFD group showed enhanced activity in FXII, FVIII and FVII (Fig. 7).

## Discussion

The object of the present study was to compare control and experimental diets used in obesity studies. We found that both CAF and HFD led reliably to obesity. The body weight in HFD and CAF mice was significantly higher than that of SCD and NFD mice, which were comparable in this regard. This is consistent with previous studies^4,6^.

Some studies have reported that CAF feeding resulted in a more pronounced obesity than HFD in Wistar rats^6,25^ and in BALB/c mice^4^. This was not observed in the present study; no significant effects on adiposity were found between CAF and HFD, nor between SCD and NFD. This discrepancy between our study and previous studies may result from different energy intakes. While the daily energy intake in the CAF group was reported to be higher than in the HFD group^4,6,25^, we observed no significant difference in daily energy intake between CAF and HFD groups (Table 1). The reason for this could be in the detailed composition and taste of CAF and HFD used. Surprisingly, the weight of adipose tissues was reported to be similar between the NFD and HFD groups in one of the above studies^6^. In our study, in contrast, the fat mass in both the DIO groups (CAF and HFD) was clearly greater than both of the control groups (SCD and NFD) (Fig. 2).

Impaired glucose tolerance and insulin resistance are the most important pathologies in type 2 diabetes mellitus and metabolic syndrome. Thus, a reliable induction of impaired glucose tolerance is an essential feature of high caloric diets used in animal studies. In the present study, hyperglycemia and impaired glucose tolerance were found in both DIO groups (HFD and CAF) within twelve weeks of feeding. The mice of both groups showed increased fasting glucose values, an increased AUC of the GTT and 2-hour-values in the GTT. In addition, decreased insulin sensitivity could be observed in both HFD- and CAF-fed mice. Whereas we found no difference between CAF and HFD, a previous study demonstrated a higher efficiency of CAF to induce hyperglycaemia as well as an earlier induction of glucose intolerance and insulin resistance by CAF compared to HFD in C57BL/6J mice^18^. A greater insulin resistance after CAF in comparison to HFD was also reported by Sampey *et al*. in rats^6^. This may be due to the higher visceral adiposity of the rats on CAF compared to HFD observed in that study^6^. There was no significant difference between the control groups in terms of fasting glucose, results of GTT and ITT and plasma insulin levels (Fig. 3). According to these results, both control groups can be considered comparable and reliable control diets for studies investigating impaired glucose tolerance.

Obesity is an important underlying factor of vascular dysfunction. A central mechanism in vascular dysfunction is attributed to the reduction of endothelial NO bioavailability. Reduced NO bioavailability may be due to enhanced NO inactivation by superoxide or because of decreased NO production as the consequence of eNOS uncoupling and/or eNOS inhibition^26–28^. Besides the commonly known endothelial dysfunction in obesity, recent studies have shown that the PVAT plays an important role in obesity-induced vascular dysfunction^29–31^. The PVAT of healthy animals attenuates agonist-induced vasoconstriction^32^. In the vessels of obese mice, however, PVAT worsens vascular function^20,21,33^. Similar observations have been made in human vessels in *in vitro* studies^34^.

In the present study, no difference in the acetylcholine-induced relaxation of the PVAT-free aorta was observed between any of the groups (Fig. 4). This observation is consistent with the findings of previous studies showing that PVAT plays a more important role than the endothelium in obesity-induced vascular dysfunction^20,21,29^. A clear reduction of acetylcholine-induced vasodilation could be observed in PVAT-containing aorta from HFD-fed mice^20,21,33^. In these studies, the mice were treated with HFD for 18 weeks^21^, 22 weeks^20^ and 8 months^33^, respectively. In the present study, the mice were put on diets only for 12 weeks. At this early stage, vascular dysfunction was insufficiently developed, yet. Nevertheless, it was evident that vascular function was best in the NFD group and worst in the CAF group, with NFD > SCD > HFD > CAF (Fig. 4). In addition to PVAT and endothelial cells, vascular smooth muscle cells (VSMCs) also represent a relevant source of NO in the vasculature and thereby play a role in regulating vascular tone. The expression of inducible NO synthase (iNOS) can be upregulated through transcriptional and posttranscriptional mechanisms in VSMCs^35^, e.g. by proinflammatory cytokines^36^. Obesity is associated with chronic low-grade inflammation^37^, and iNOS expression was shown in diet-induced obese mice^38^ and rats^39^. Moreover, deficiency of iNOS led to an amelioration of diet-related insulin resistance^40,41^. Therefore, a potential contribution of VSMC iNOS to vascular dysfunction in DIO mice should be investigated in future studies.

The acetylcholine-induced vasodilation in PVAT-containing aorta could be completely prevented by the non-selective eNOS inhibitor L-NAME (Fig. 4) in all groups, indicating that this vasodilator response is mediated solely by NO. This is consistent with previous studies^20,21^. Indeed, our previous studies have demonstrated that eNOS dysfunction in the PVAT is a crucial mechanism underlying obesity-induced vascular dysfunction, with reduced PVAT eNOS phosphorylation as a contributing mechanism^20,21^. Consistently, the present study shows that both DIO diets (HFD and CAF) led to a marked reduction of PVAT eNOS phosphorylation at serine 1177 and reduced phosphorylation of the upstream kinase Akt (Fig. 5).

Obesity is an established risk factor for thrombotic disorders and is associated with increased risk of ischemic stroke, deep vein thrombosis, and pulmonary embolism^42,43^. In the present study, both aPTT and PT were shortened in HFD mice compared to the SCD group (Fig. 6), indicating higher activity of both the intrinsic and extrinsic coagulation pathway. This is consistent with previous studies showing that HFD-fed C57BL/6J mice exhibited significantly shorter aPTT and PT than lean mice fed a standard diet^44^. HFD-feeding of C57BL/6J mice results in higher thrombus weight in a thrombosis model^45^ and to increased plasma levels of coagulation factors FII, FVII, FVIII, FIX, FXI and FXII^46^. This may be part of the mechanisms underlying the enhanced activity of the individual coagulation factors observed in the present study (Fig. 7).

Animal studies addressing coagulation and thrombosis in which CAF was used could be hardly found. In one study, CAF feeding of Wister rats for 8 weeks led to a shortening of aPTT without any effect on PT^47^. In another study, 24 weeks CAF feeding of rats showed no effects on PT and aPTT compared to SCD^48^.

This is the first study comparing different DIO diets for their effects on the coagulation system. Interestingly, we found significant difference between NFD and SCD, especially regarding coagulation (Figs. 6 and 7). Shortened PT and aPTT in NFD could be due to the higher amount of carbohydrates in the diet. Carbohydrates were found to induce lower aPTT as well as Russel viper venom clotting time^49^. Furthermore, various studies analysing the effect of hyperglycaemia on coagulation have demonstrated a connection *in vitro* and *in vivo*^50^. Endothelial cells from pig aortas treated with high concentrations of glucose let to an increase of plasminogen activator inhibitor-1 and correspondingly to an inhibition of fibrinolysis^51^. In humans, hyperglycaemia led to coagulation activation characterized by increased levels of soluble tissue factor and elevated levels of thrombin-antithrombin complexes^52^. The combination of hyperglycaemia and hyperinsulinemia in humans showed the highest coagulation activity accompanied by raised numbers of thrombin-antithrombin complexes, FVII, FVII, activated platelets as wells as soluble tissue factor procoagulant activity^53^. Consequently, these results raise the question, what is the correct control diet in obesity studies.

The choice of the correct control diet for HFD is subject of controversial discussions^11,13,14,54^. Whereas a large number of studies uses SCD as control diet, others choose NFD and some studies do not even clarify the constituents of the used control diet^10^. SCD is a low-cost and in the most laboratories an easily accessible diet type, which is also used in diet-unrelated studies. It usually is grain-based and further consists of corn, wheat, soybean, alfalfa or oat^14^. The exact combinations are not fixed leading to differences in nutrient levels from batch to batch^12,14^. NFD, however, is available with the exact identical ingredients as HFD with exception of the fat derived energy of HFD, which is replaced with sugar in NFD^11^. This way, the differences between the consumed food of the control and obesity groups are limited to the energy content which also enhances reproducibility^13^. However, assuming NFD is the superior diet type is premature. Since NFD contains high amounts of sugar and low fiber, a study using NFD as control for HFD compares a high-fat diet with a high-sugar diet and not with a normal diet^11,55^.

In this work, several differences between SCD- and NFD-fed mice were observed. They ranged from food consumption and lumbar fat mass to vascular function and coagulation activity. Thus, these diet forms are not only different regarding their ingredients and nutrition facts, but also in terms of the typical end points of DIO studies. Following this, SCD and NFD should not be considered equal. This finding is in line with previous studies which focussed especially on the effects of the content of fibre in NFD^55^. It was shown that, in dependency of the level and type of fibre in a diet, gut morphology and microbiome varied in mice^12^. Moreover, the lack of soluble fibre was correlated with cecal and colonic atrophy that resulted in microbiota-dependent promotion of adiposity^16^. In CB57BL/6 mice, these effects were confirmed for NFD by Dalby *et al*.^56^ and explained by Chassaing *et al*. as a result of lack of soluble fibre in the diet^16^.

The correct control type depends on the question to be answered with the respective study^54^. If the effect of a diet rich in fat is subject of the study, NFD should be used. If the effect of high energy intake shall be compared to a healthy population, SCD can be considered the appropriate control. There might be certain studies investigating the effect of high fat intake in particular. But the predominant part of studies using HFD to achieve DIO intend to investigate the effect of high caloric intake, as it happens in western society compared to a healthy control group. In such cases, it seems acceptable to use SCD, which is also used in non-diet related studies to provide animals without diet-derived pathologies. It is of upmost relevance that every study dealing with experimental feeding publishes the exact composition and the nutrition facts of the used SCD to enable comparability. Furthermore, there is a necessity to raise awareness for the importance of the correct control diet and the exact definition of the used diet types.

CAF feeding reliably induced visceral obesity, glucose intolerance, insulin resistance and vascular dysfunction in the present study. These effects were comparable to those in 45% HFD-fed mice. Moreover, regarding vascular dysfunction, the effect of CAF was even more pronounced than the one from 45% HFD (Fig. 4). Similarly, a previous study has also concluded that CAF is a robust model of human metabolic syndrome (Sampey *et al*., 2011). Nevertheless, an obvious downside of CAF is the poor comparability of the studies. There is currently no standardization available. The used snacks in the different studies are not always available in every country. Therefore, the composition of food and energy amount consumed by the animals fed with CAF can vary substantially. Concerning these factors, as well as the higher personal resources needed to maintain a daily changing food supply (compared to HFD, which usually needs only to be changed weekly or twice a week), we do not suggest CAF as a standard method in obesity research. It is however a valuable special diet, if the aim of a research is to investigate the behavioural, addiction-like aspects of highly palatable food and its effect on the development of metabolic syndrome.

The present study is supposed to serve as a helpful tool and an appeal for decision making for choosing the right diet for experiments concerning DIO-induced metabolic disorders. Nevertheless, our study has several limitations. First, all experiments were performed with male mice only. However, obesity influences males and females differently since sex hormones affect body adiposity as well as the metabolic system^57^. To address this sexual dimorphism, future experiments with female mice are needed. Second, anaesthesia before organ isolation was performed with isoflurane. Isoflurane is a known vasodilator^58^ and therefore one could argue that the performed measurements for vascular function assessment may be biased. But since all animal groups were anesthetized with isoflurane the comparison between the groups is still valid. Third, an additional approach to analyse insulin resistance is to perform vascular relaxation experiments with insulin as a vasodilator^59^, what was not done in the present study.

## Conclusion

CAF and HFD are both reliable mouse diets in inducing visceral obesity, glucose intolerance and insulin resistance. Standard chow and NFD are comparable in this regard. CAF is more effective than HFD in causing PVAT dysfunction and vascular dysfunction, whereas hypercoagulability was mostly evident in the HFD group. Coagulation activity was higher in the NFD control group than in the chow control group.

## Data Availability

The datasets used and/or analyzed in the current study are available from the corresponding author on reasonable request.
